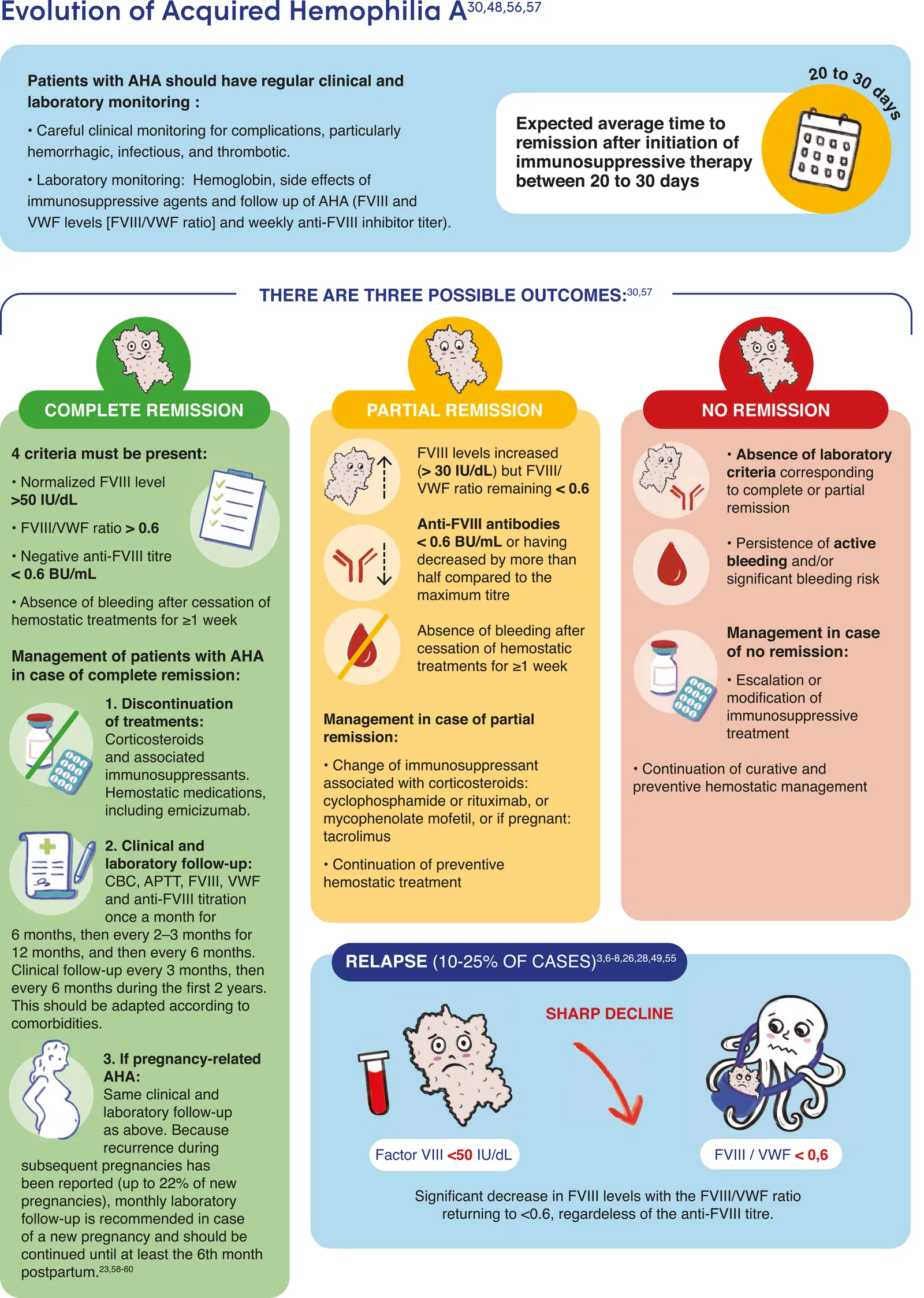# Acquired hemophilia A: an illustrated review based on the French National Guidelines

**DOI:** 10.1016/j.rpth.2026.106820

**Published:** 2026-07-27

**Authors:** Benoît Guillet, Roseline d’Oiron, Sebastien Lacroix-Desmazes, Valerie Chamouard, Achille Aouba, Sabine Castet, Ygal Benhamou, Christophe Nougier, Nicolas Noel, Yohann Repesse, Julie Graveleau, Marc Trossaert, Hervé Lévesque, Yesim Dargaud

**Affiliations:** 1 Centre de référence de l’hémophilie et des maladies hémorragiques, constitutionnelles, Centre Hospitalier Universitaire de Rennes, Rennes, France; 2 École des hautes études en santé publique, Institut de recherche en santé, environnement et travail (IRSET), Centre Hospitalier Universitaire de Rennes, université de Rennes, Inserm, Rennes, France; 3 Centre de référence de l’hémophilie et des maladies hémorragiques, constitutionnelles, hôpital Bicêtre, Assistance Publique–Hôpitaux de Paris, Unite de Recherche Inserm 1176 Hémostase, Inflammation, Thrombose, Inserm, Université Saclay, Le Kremlin-Bicêtre, Paris, France; 4 Institut National de la Santé et de la Recherche Médicale, Centre de Recherche des Cordeliers, CNRS, Sorbonne Université, Université de Paris, Paris, France; 5 French Reference Center for Hemophilia, Hospices Civils de Lyon, University Claude Bernard Lyon 1, Lyon, France; 6 Service de médecine interne, Centre Hospitalier Universitaire de Caen, Caen, France; 7 Centre de Ressources et de Compétences de la filière MHEMO, Centre Hospitalier Universitaire de Bordeaux, Bordeaux, France; 8 Service de médecine interne, Centre Hospitalier Universitaire de Rouen, Normandie université, UNIROUEN, 1, rue de Germont, Rouen, France; 9 Service de médecine interne hôpital Bicêtre, Assistance Publique–Hôpitaux de Paris, Le Kremlin-Bicêtre, Paris, France; 10 Centre de Ressources et de Compétences de la filière MHEMO, Centre Hospitalier Universitaire de Caen, Caen, France; 11 Service de médecine interne, Centre Hospitalier Universitaire de Nantes, Nantes, France; 12 Centre de Ressources et de Compétences de la filière MHEMO, Centre Hospitalier Universitaire de Nantes, France

**Keywords:** acquired hemophilia A, bypassing therapy, emicizumab, inhibitor, porcine factor VIII

## Abstract

Acquired hemophilia A is a rare but potentially life-threatening autoimmune bleeding disorder caused by the sudden development of neutralizing autoantibodies against factor VIII (FVIII), predominantly affecting older adults. Because of the risk of severe bleeding and excess mortality, prompt hospitalization and immediate management are essential to avoid diagnostic and therapeutic delays. Diagnosis relies on the association of an isolated prolonged activated partial thromboplastin time, reduced FVIII activity, and detection of a FVIII inhibitor quantified using the Bethesda or Nijmegen assay.

Management has 2 complementary objectives. First, hemostatic therapy combines preventive measures with treatment of acute bleeding episodes, using bypassing agents (recombinant activated FVIII or activated prothrombin complex concentrate) or recombinant porcine factor VIII as first-line options.

More recently, prophylaxis with emicizumab has emerged as a promising strategy to reduce recurrent bleeding, with early clinical data suggesting favorable efficacy and safety. Second, inhibitor eradication relies on immunosuppressive therapy, typically corticosteroids alone or in combination with agents such as cyclophosphamide or rituximab, guided by baseline FVIII activity and inhibitor titer.

Close and prolonged follow-up is mandatory, combining clinical assessment of bleeding control, recurrence risk, and treatment toxicity, with laboratory monitoring of hemoglobin, factor VIII activity, von Willebrand factor levels, and inhibitor titers. Surveillance should be maintained for at least 2 years after complete remission.

**Figure:**
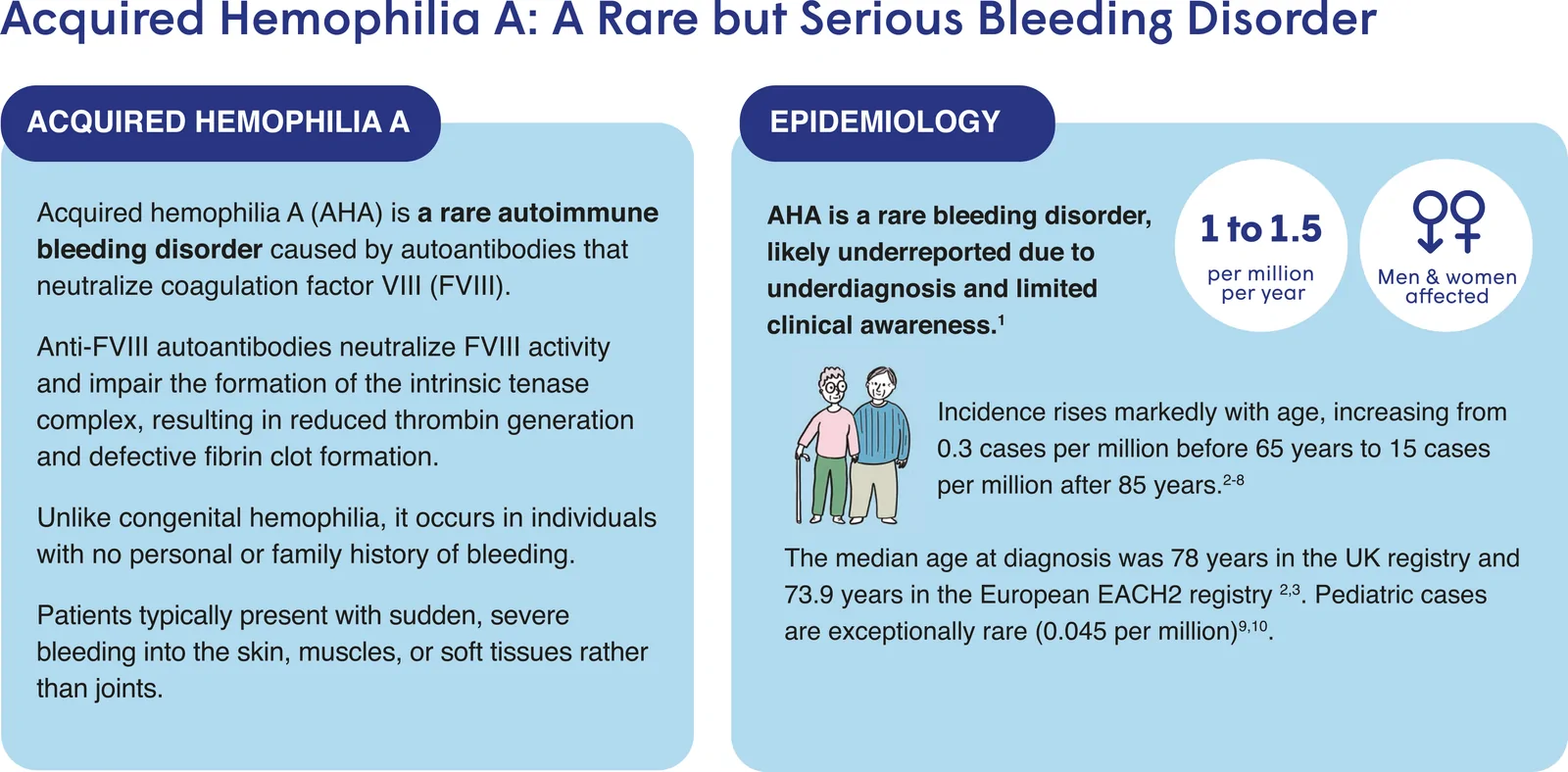

**Figure:**
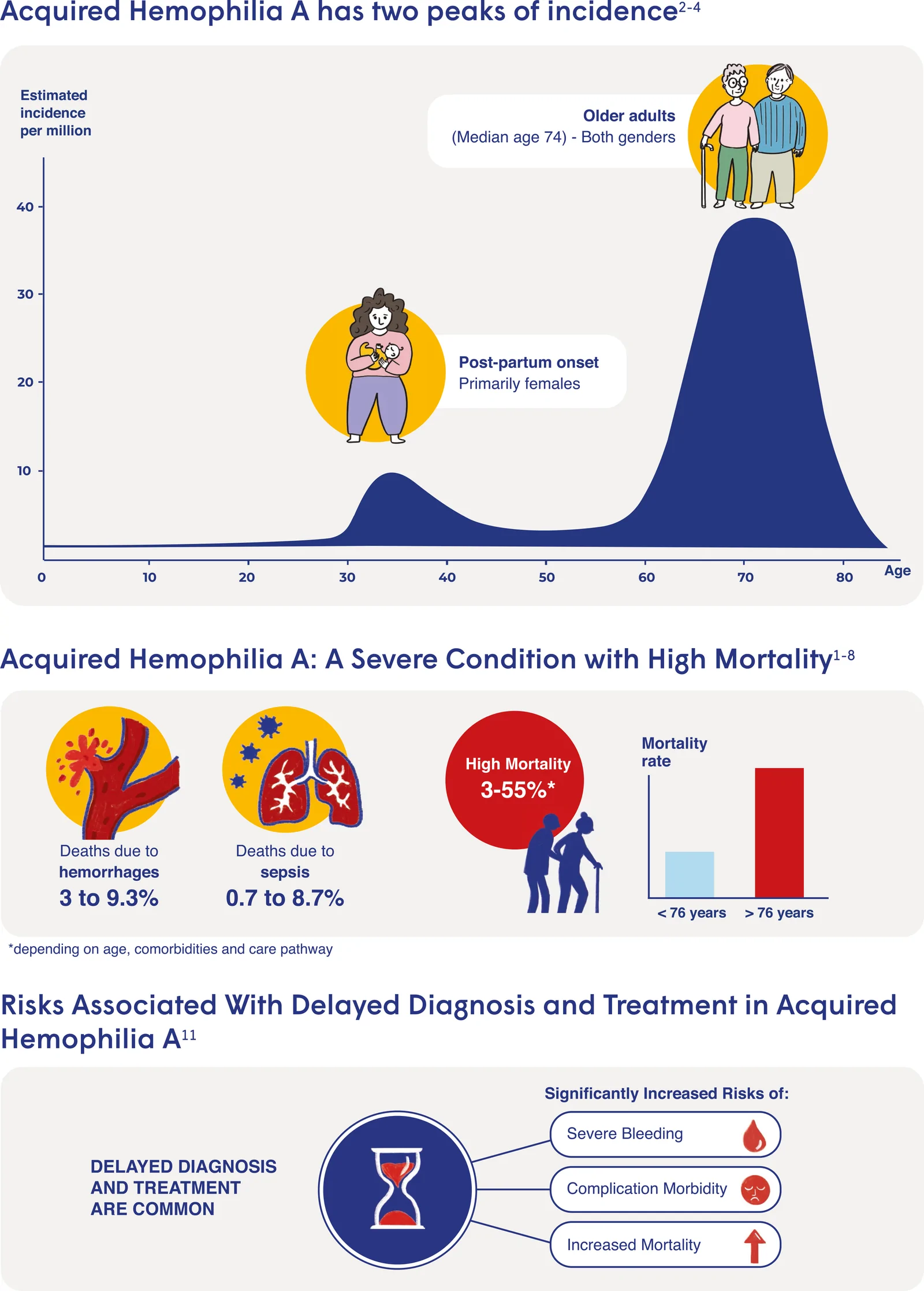

**Figure:**
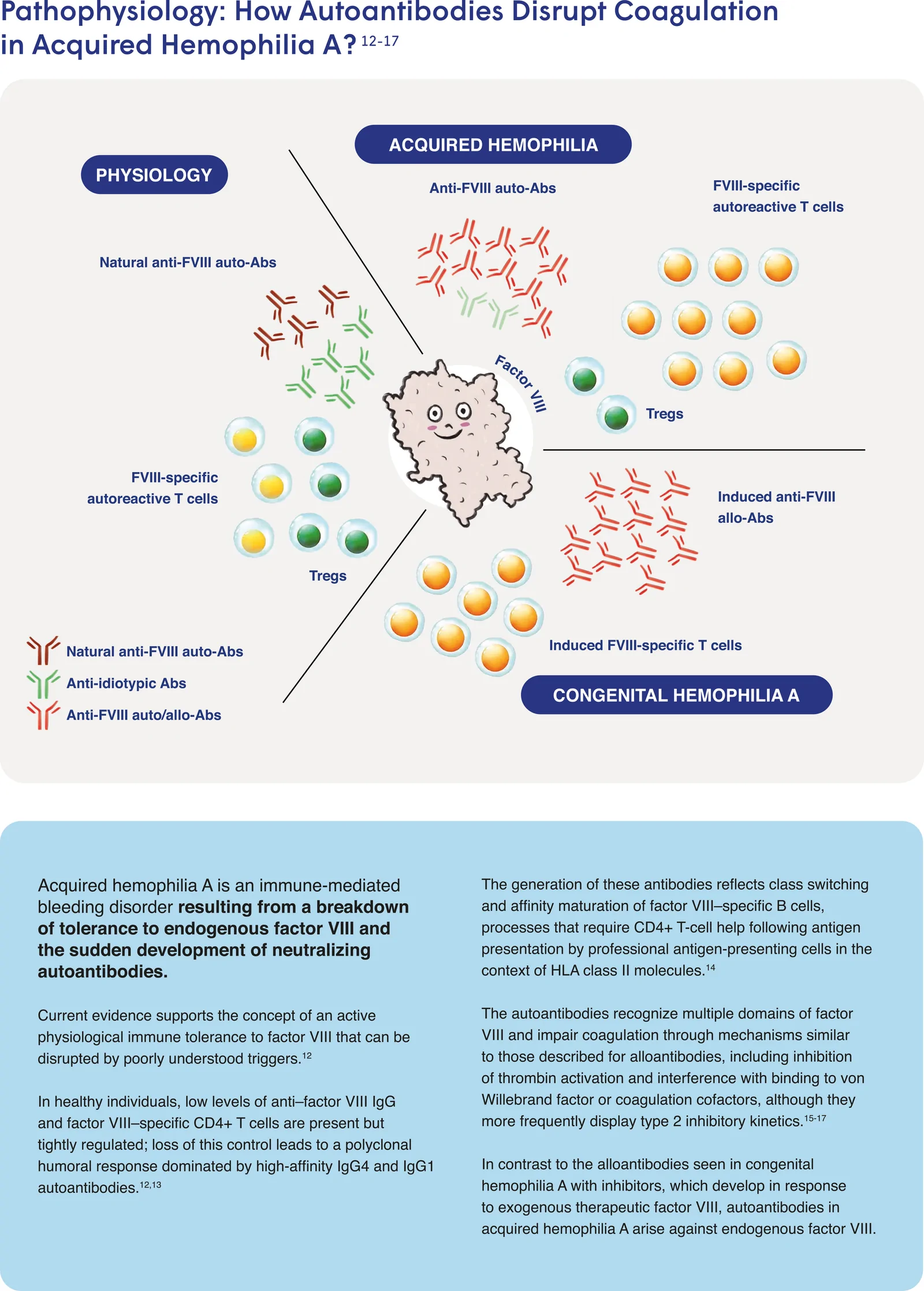

**Figure:**
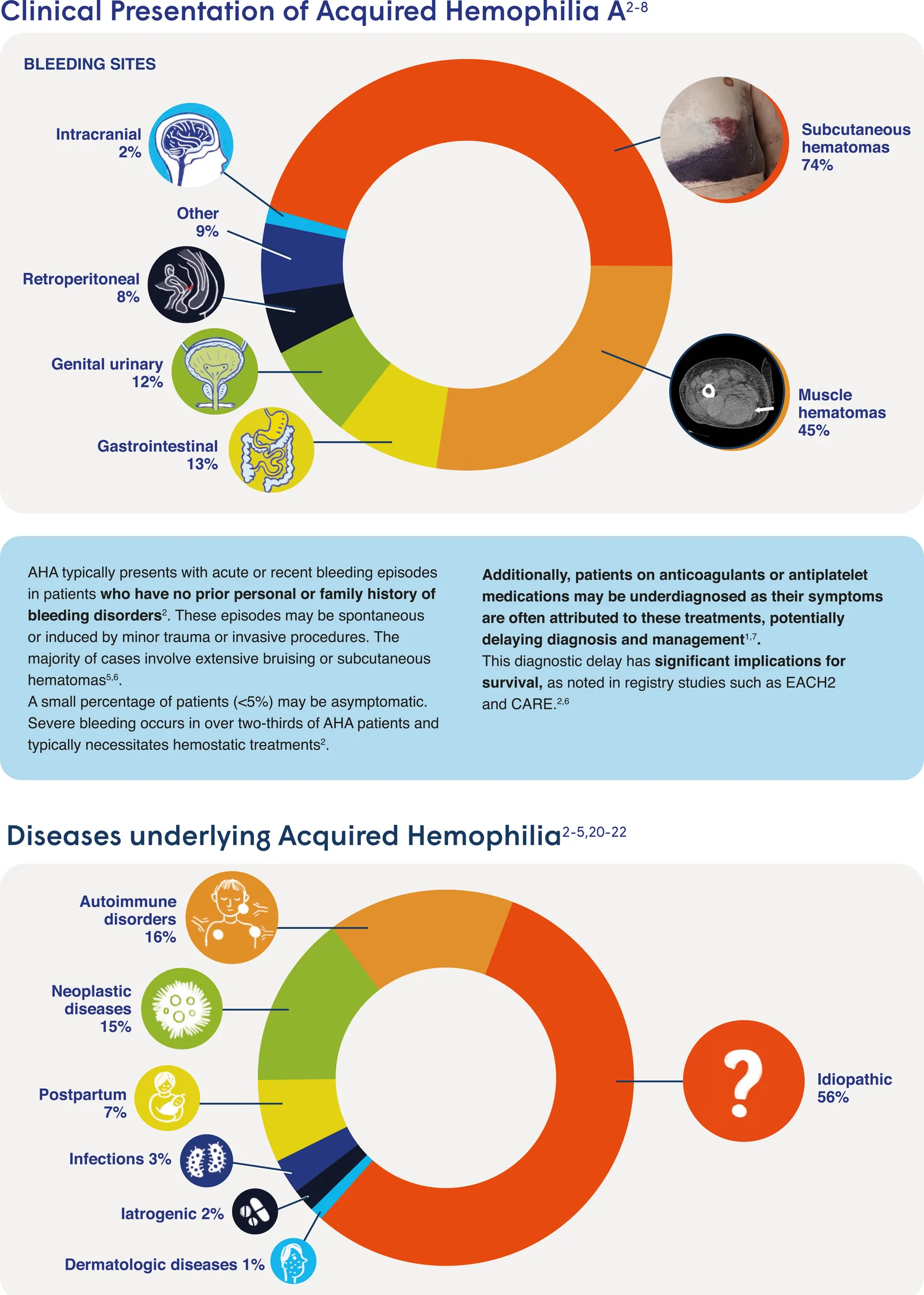

**Figure:**
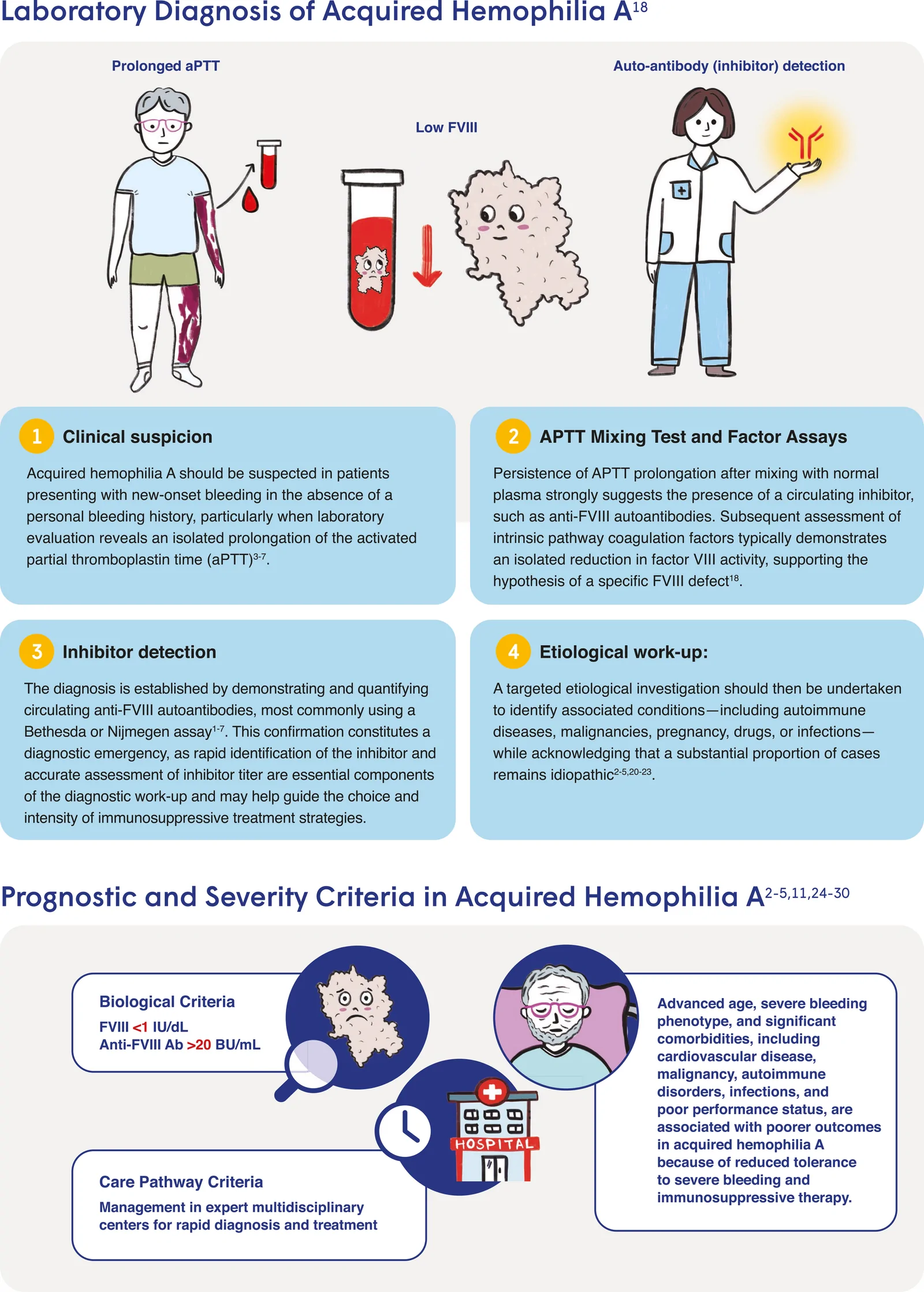

**Figure:**
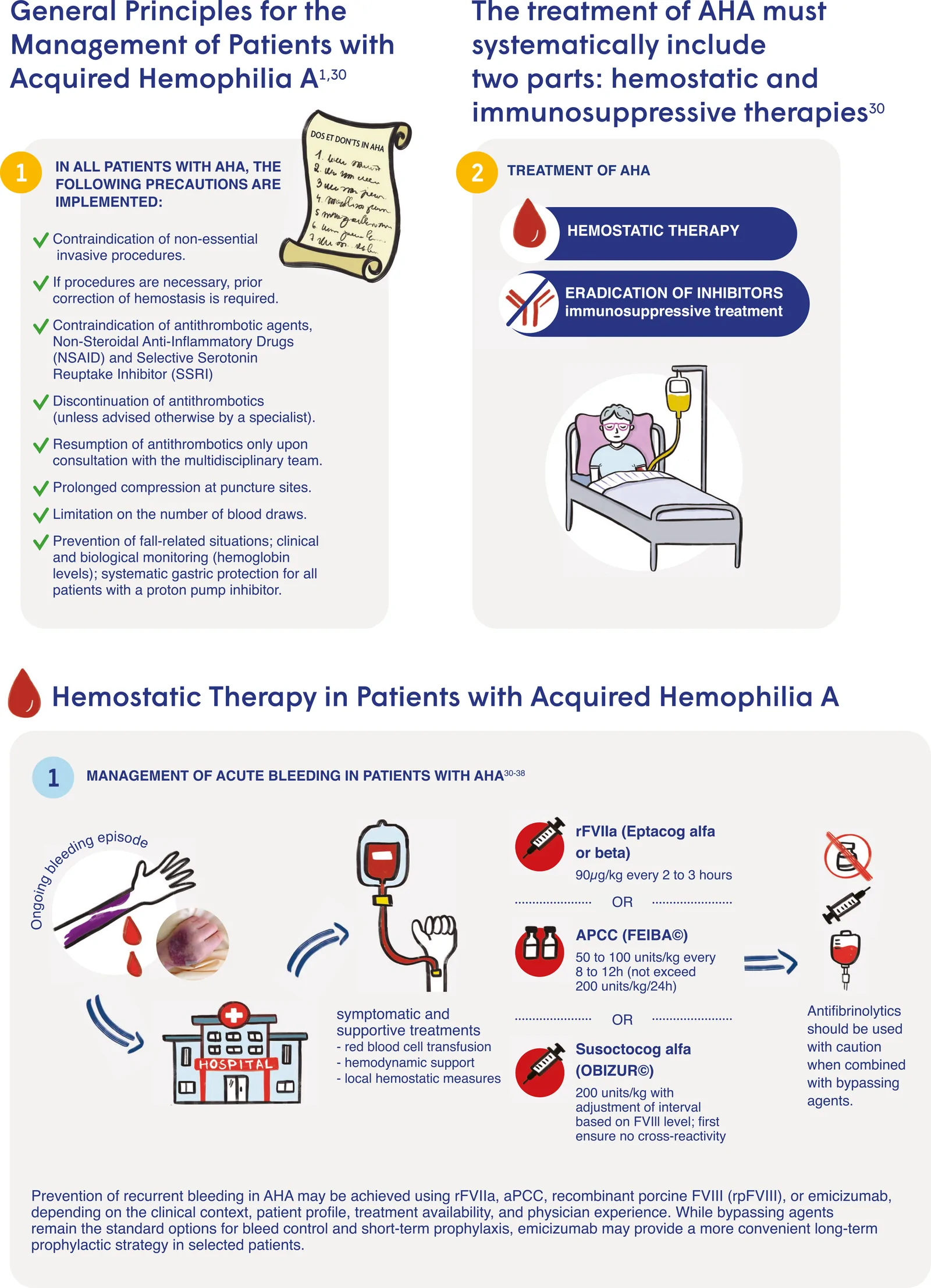

**Figure:**
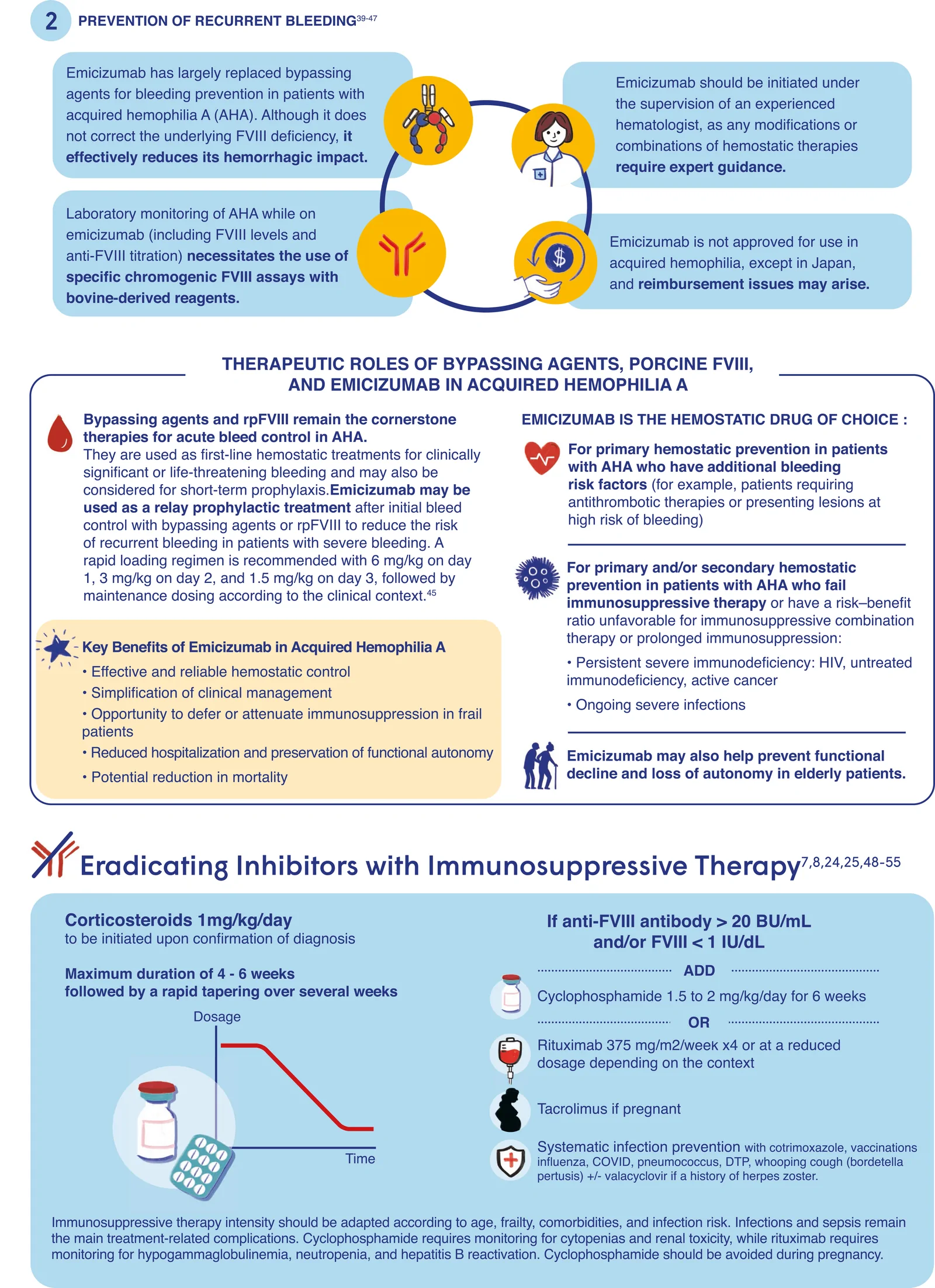

**Figure:**